# Predictive modeling of *Salmonella* Thompson growth in unpasteurized liquid egg products

**DOI:** 10.5713/ab.250655

**Published:** 2026-02-06

**Authors:** Chang-Geun Lim, Seung-Hee Baek, In-Sik Nam

**Affiliations:** 1 Department of Animal Convergence Science, Hankyoung National University, Anseong, Korea; 2 Research Center for Environment Friendly and Quality Livestock Production Technology, Hankyoung National University, Anseong, Korea; 3 Institute of Applied Humanimal Science, Hankyong National University, Anseong, Korea

**Keywords:** Food Safety, Growth Kinetics, Predictive Modeling, *Salmonella* Thompson, Unpasteurized Liquid Egg

## Abstract

**Objective:**

*Salmonella* Thompson is a major cause of large-scale foodborne disease outbreaks worldwide; however, research on *S*. Thompson remains limited. This study investigates the development of a predictive model for the growth of S. Thompson in unpasteurized liquid egg products, such as liquid egg white, liquid egg yolk, and liquid whole egg, to understand the associated health risks.

**Methods:**

Unpasteurized liquid egg products, confirmed to be free of *Salmonella* spp., were inoculated with *S*. Thompson and incubated at various temperatures. Growth kinetic parameters were estimated using both primary and secondary predictive models, including the Baranyi and Roberts model and second-order polynomial models. The effects of environmental factors on *S*. Thompson growth were analyzed to establish a comprehensive risk assessment framework.

**Results:**

The growth curves of *S*. Thompson exhibited a typical bacterial sigmoidal pattern characteristic of bacterial proliferation, with the Baranyi model providing the best fit for describing the growth kinetics. The secondary model accurately predicted the effect of temperature on growth rate, demonstrating that *S*. Thompson proliferates rapidly under specific environmental conditions. Model validation indicated high accuracy, confirming the reliability of the developed model for risk assessment applications.

**Conclusion:**

The established predictive model enables quantitative assessment of the growth behavior of *S*. Thompson in unpasteurized liquid egg products. This model can be used in risk assessment and food safety management strategies to mitigate the risk of foodborne pathogen contamination in the food industry.

## INTRODUCTION

*Salmonella* is one of the most common pathogens responsible for foodborne diseases worldwide [1,2]. Every year, *Salmonella* causes approximately 93.8 million cases of gastroenteritis globally, resulting in an estimated 155,000 deaths. In Korea, reported outbreaks of gastroenteritis have steadily increased from 261 cases in 2013 to 422 cases in 2015 and 544 cases in 2017. Of the 544 cases reported in 2017, pathogens were identified in 294 cases (54.0%), with *Salmonella* detected in 23 cases (7.8%) [3]. These data highlight the public health significance of *Salmonella* infections in both developed and developing countries [4].

*Salmonella* is commonly transmitted through chicken meat and eggs owing to its ability to contaminate eggs [5]. Toyofuku [6] reported that most salmonellosis cases in Japan between 1998 and 2004 were associated with egg consumption. Similarly, Harker et al [7] identified consumption of contaminated eggs as the primary cause of salmonellosis outbreaks in England and Wales between 2000 and 2011. According to an epidemiological investigation of salmonellosis cases in South Korea in 2023, approximately 20% of infections were linked to egg consumption [8].

As egg consumption has diversified in recent years, refrigerated liquid eggs have become increasingly popular. Liquid eggs are widely used in the baking industry and in institutional food service systems [9]. These products are typically consumed as unpasteurized, raising safety concerns regarding foodborne pathogens. Over 2.7 billion pounds of liquid egg products are sold annually in the United States [10]. However, some of these products have been associated with foodborne illnesses caused by *Salmonella* [11]. There have also been cases of *Salmonella* infection linked to the consumption of liquid egg products in England and Ireland [12]. The United States classifies liquid eggs as a high-risk food associated with foodborne infections [11]. In South Korea, a large-scale food poisoning outbreak involving 2,207 cases occurred due to chocolate cake served in school meals. The outbreak was traced to *Salmonella* Thompson contamination of liquid egg whites (LEWs) used as an ingredient in the cake [3,13]. This highlights the significance of understanding the emerging risks of *S.* Thompson for effective control of foodborne pathogens.

Quantitative microbial risk assessment is used to evaluate the risk of infection due to exposure to microorganisms, thereby aiding in the management of microbial food safety risks and reducing the frequency of foodborne outbreaks [14]. Currently, most studies on foodborne illnesses focus on quantitative risk assessments, using mathematical tools from predictive microbiology to evaluate risks [15]. Kim et al [16] modeled five types of *Salmonella* in egg products, whereas Singh et al [17] studied *Salmonella* spp. in whole liquid eggs. Research on *Salmonella* in eggs has primarily focused on the serotypes associated with major zoonotic infectious diseases, such as *S.* Enteritidis and *S.* Typhimurium. However, despite being a major cause of large-scale foodborne disease outbreaks, research on *S.* Thompson remains limited.

The purpose of this study was to analyze the growth dynamics of *S.* Thompson in unpasteurized liquid egg products and to develop and validate a predictive growth model. We investigated the growth of *S.* Thompson under various temperature conditions and quantified the associated risks posed by temperature fluctuations. The findings can inform risk management decisions regarding the storage time and temperature of unpasteurized liquid egg products. Furthermore, this study provides valuable insights into the growth characteristics of *S.* Thompson.

## MATERIALS AND METHODS

### Sample preparation

The LEW, liquid egg yolk (LEY), and liquid whole egg (LWE) used in this study were unpasteurized, commercially available products purchased from a major online retail market in Korea throughout the experimental period. To reflect typical consumer products, items were selected among the most frequently purchased liquid egg products on the platform. All samples were stored in a refrigerator at 4°C and used before the manufacturer’s expiration date. To verify sample contamination, a *Salmonella* spp. qualitative test was conducted according to microbial testing methods outlined in the Korean Food Code. A 25 mL sample was added to 225 mL of Buffered Peptone Water (MB Cell) and incubated at 36±1°C for 24 h. Briefly, 1 mL and 0.1 mL of the cultured Buffered Peptone Water were added to 10 mL of Tetrathionate broth (MB Cell) and 10 mL of Rappaport-Vassiliadis broth (MB Cell), respectively. These were then incubated at 36±1°C and 41.5±1°C for 24 h, respectively. Each culture was smeared on Xylose Lysine Deoxycholate agar (XLD; MB cell) and incubated at 36±1°C for 24 h.

### Bacterial inoculation

*S.* Thompson strain NCCP 11704 was obtained from the National Culture Collection for Pathogens (Korea). *S.* Thompson was cultivated twice in Tryptic Soy Broth (TSB; Difco) at 37°C for 24 h. The resulting cell suspension was then transferred to Tryptic Soy Agar (MB Cell) and incubated at 37°C for 24 h. Subsequently, a 10 mL aliquot of liquid egg was placed into a 50 mL sterile tube and inoculated with *S.* Thompson at an initial concentration of 5 log CFU/mL. The inoculated liquid egg samples were then stored at 5°C, 15°C, 25°C, and 35°C. At each temperature and time point, 1 mL was withdrawn from each sample and serially diluted in saline using decimal dilution. The diluted solutions were then dispensed into XLD agar, a selective medium for, and cultured at 37°C for 24 h. After incubation, the bacterial colonies were counted and expressed as log CFU/mL.

### Modeling

The primary predictive growth model (Eqs. 1, 2) was based on the Baranyi model introduced by Baranyi and Roberts [18]. Parameters such as lag time (LT; h) and maximum specific growth rate (*μ*_max_; log CFU/mL/h) were estimated by fitting the experimental growth data to this model using DMFit software, a tool provided by ComBase (Institute of Food Research). The Baranyi model is expressed as follows:


(1) $$y_{t}=y_{0}+\mu_{max}F(t)-ln\left(1+\frac{exp^{\mu_{max}f(t)}-1}{exp^{\left(y_{max}-y_{0}\right)}}\right)$$

(2) $$F(t)=t+\frac{1}{\nu }ln\left(e^{-\nu t}+e^{-h_{0}}-e^{\left(-\nu t-h_{0}\right)}\right)$$

(i) *y*_t_: cell density at time (t); (ii) *y*_0_: initial cell density; (iii) *y*_max_: maximum cell density; (iv) *μ*_max_: specific growth rate; (v) *v*: rate of increase in the limiting substrate (assumed to be equal to *μ*_max_); (vi) *h*_0_: calculated by multiplying the *μ*_max_ by the lag time.

The secondary model describes the effects of temperature on the parameters estimated by the primary model. Second-order polynomial models (Eq. 3) [19,20] were employed to analyze the effects of temperature on the specific growth rate (*μ*_max_) and lag time (*LT*), as calculated from the primary models using GraphPad Prism 9.0 (GraphPad Software).


(3) $$LT\&\mu_{\text{max}}=b_{0}+(b_{1}\times T)+(b_{2}\times T^{2})$$

(i) *b*_0_, *b*_1_, *b*_2_: regression constants; (ii) *T*: temperature.

### Validation

The effectiveness of the growth prediction model was evaluated by fitting the *S.* Thompson growth curves in liquid eggs to the Baranyi model. To assess the performance of both the primary and secondary models, three statistical indices were used: bias factor (B*_f_*), accuracy factor (A*_f_*), and root mean square error (RMSE), as represented by Eq. 4 [21]. The model was considered acceptable when B*_f_* values ranged between 0.7 and 1.15, A*_f_* was close to 1, and RMSE values were low, indicating high predictive accuracy for *S.* Thompson growth.


(4) $$B_{f}=10\frac{\sum log\left(\frac{Pred}{Obs}\right)}{n}$$

(5) $$A_{f}=10\frac{\sum \left|log\left(\frac{Pred}{Obs}\right)\right|}{n}$$

(6) $$RMSE=\sqrt{{\sum \left(\frac{Obs-Pred}{n}\right)}^{2}}$$

(i) *Obs*: observed values; (ii) *Pred*: predicted values; (iii) *n*: representing the number of observations.

### Statistical analysis

All experiments were conducted in triplicates. All statistical analyses were conducted in IBM (SPSS 21.0) using Duncan’s multiple range test. Duncan’s test was used to determine differences between the mean values of the pathogens, sampling times, and temperatures. Differences were considered statistically significant at p≤0.05.

## RESULTS AND DISCUSSION

Unpasteurized liquid egg products confirmed to be free of *Salmonella* spp. contamination by qualitative testing were used in the present study. The growth dynamics of *S.* Thompson in these products were examined across various temperature ranges. Based on the findings, a predictive growth model was developed and validated.

### Growth characteristics of *Salmonella* Thompson

**Figure 1:**
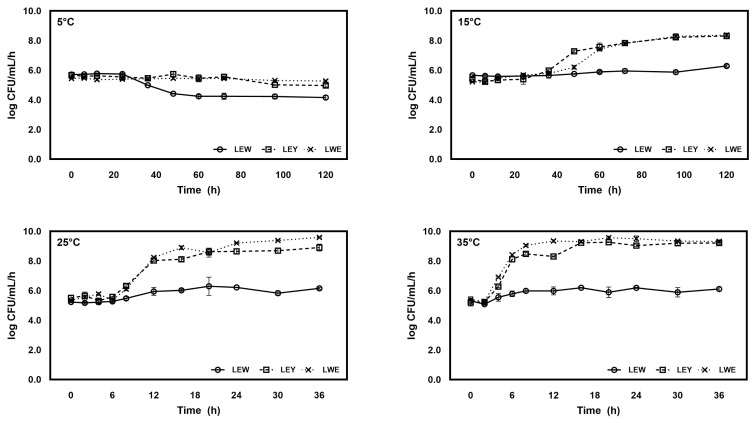
Growth of *Salmonella* Thompson in unpasteurized liquid egg whites, liquid egg yolks, and liquid whole eggs at different temperatures. The means of three replicates are plotted with the error bars representing the standard deviation. LEW, liquid egg white; LEY, liquid egg yolk; LWE, liquid whole egg.

The growth of *S.* Thompson at 5°C, 15°C, 25°C, and 35°C in LEW, LEY, and LWE is shown in Figure 1. The initial cell density was approximately 5.39±0.16 log CFU/mL under all conditions. No growth of *S.* Thompson was observed at 5°C in any sample (Figure 1A). When stored at 15°C, *S.* Thompson exhibited slight growth in all samples (Figure 1B). The final cell density reached 6.28±0.08 log CFU/mL in LEW, 8.30± 0.19 log CFU/mL in LEY, and 7.42±0.05 log CFU/mL in LWE, indicating significant differences in growth among the samples (p<0.05). As the temperature increased, the growth of *S.* Thompson progressed gradually, with LEW exhibiting noticeable growth. After storage at 25°C, the final cell densities of *S.* Thompson were found to be 6.15±0.07 log CFU/mL in LEW, 8.91±0.24 log CFU/mL in LEY, and 9.59±0.04 log CFU/mL in LWE (Figure 1C). At 35°C, the final cell densities of *S.* Thompson were 6.11±0.13 log CFU/mL in LEW, 9.21±0.11 log CFU/mL in LEY, and 9.32±0.11 log CFU/mL in LWE (Figure 1D). Egg whites are widely acknowledged as a markedly more restrictive medium for microbial growth than whole eggs or egg yolks [22,23]. The lack of growth of *Salmonella* in LEW may result from the combined effects of microbial inhibitors, such as ovotransferrin, ovomucin, cystatin, and avidin, which are present in egg products [10,24,25]. In contrast, LEY and LWE created a nutrient-rich environment that was conducive to the growth of *Salmonella* [22]. Our findings also revealed that the growth of *S.* Thompson was restricted by temperature in LEW, whereas rapid growth was observed in both LEY and LWE.

### Primary model for growth

The growth kinetics of *S.* Thompson was assessed using the Baranyi model (Table 1). Baranyi and Roberts highlighted the superior ability of their model to describe bacterial growth under diverse environmental conditions [18], while Ye et al [26] demonstrated that foundational models, such as the Baranyi model, can effectively predict bacterial growth. The kinetic parameters exhibited notable changes with increasing temperatures. Minimal growth was observed in LEW as the temperature increased, with the *μ*_max_ ranging from −0.274 to 0.212 log CFU/mL/h. L Huang [27] reported that the *μ*_max_ of *S. Enteritidis* in LEW ranged from 0.081 to 0.921, which was higher than the values obtained in this study. In contrast, both the lag time (LT) and *μ*_max_ in LEY and LWE varied with rising temperatures. Notably, in LWE stored at 35°C, *S.* Thompson exhibited the shortest LT of 2.26±0.12 hours and the highest *μ*_max_ of 0.886±0.048 in LEY. This trend aligns with previous studies on *Salmonella* growth in LEYs, whole liquid eggs, and mayonnaise [28,29]. In particular, the findings of a study by Moon et al [22] reported that the LT of *S. Enteritidis* in egg yolk ranged from 3.03 to 29.23 hours, with *μ*_max_ values between 0.02 and 0.86, while *S. Typhimurium* exhibited LT values of 4.31 to 68.20 hours and *μ*_max_ values between 0.03 and 0.85. These findings suggest that *S.* Thompson follows a growth pattern similar to those described in previously developed models. No population changes were detected at 5°C or within LEW. Upon analysis using the Baranyi–Roberts model, the growth differences were not found to be significant, as indicated by the relatively low coefficients of determination (R^2^), ranging from 0.646 to 0.785. In contrast, significant population changes were observed in LEY and LWE (p<0.05), with high R^2^ values ranging from 0.946 to 0.986, demonstrating a strong model fit under these conditions. To evaluate the performance of the model, we compared the observed and predicted values using the bias factor (B*_f_*), accuracy factor (A*_f_*), and RMSE. The B*_f_* indicates whether the model’s predictions are over- or underestimated. Typically, a B*_f_* value between 0.9 and 1.05 is considered ideal, while values between 0.7 and 0.9 or 1.06 and 1.15 are considered acceptable for predictive purposes. For the A*_f_*, values closer to 1 reflect higher accuracy, and a lower RMSE approaching 0 indicates better model performance. For LEW, LEY, and LWE, the B*_f_* for *S.* Thompson ranged from 1.0005 to 1.0014, whereas the A*_f_* values ranged from 1.0170 to 1.0261, with both demonstrating a strong fit. In addition, the RMSE ranged from 0.1130 to 0.1987, further indicating that the model accurately predicted the growth of *S.* Thompson. These findings also suggest that the model can be utilized to develop risk management strategies for controlling the risk of foodborne diseases.

### Secondary model for growth

**Figure 2:**
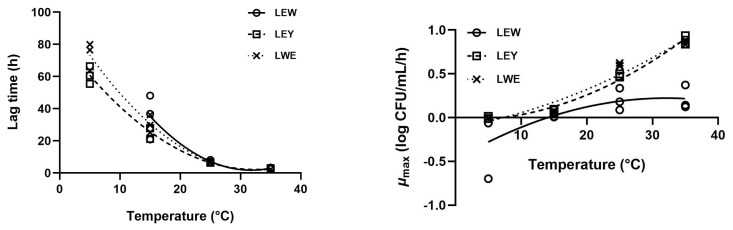
Visualization of the secondary modeling of *Salmonella* Thompson growth parameters as a function of temperature. LEW, liquid egg white; LEY, liquid egg yolk; LWE, liquid whole egg.

The secondary model was developed to describe *S.* Thompson’s LT and *μ*_max_ based on the parameters defined in the Baranyi–Roberts model. Each parameter was integrated into a second-order polynomial model (Table 2); the corresponding graphical representation is shown in Figure 2. The second-order model equations for LEY and LWE demonstrated a strong fit to the polynomial model, as reflected by high R^2^ values, ranging from 0.946 to 0.986. In contrast, while the LT for LEW also showed a robust fit, with an R^2^ value of 0.994, the *μ*_max_ displayed a comparatively lower R^2^ value of 0.578. The LT and *μ*_max_ values from the derived second-order model were comparable to those reported for *Salmonella* in egg yolk and mayonnaise [29], following a trend similar to those demonstrated via models describing the growth of *Salmonella* spp. in LWE and egg yolk [16,22]. However, in the case of egg white, the LT of *Salmonella* species was reported to be in the range of 1.16–1.55 hours at temperatures between 25°C and 35°C, with a *μ*_max_ value ranging from 0.361 to 1.192 log CFU/mL/h [28]. Guillén et al [23] reported that growth parameters may vary depending on the initial inoculum dose, and the observed differences from the findings of previous studies could be attributed to variations in the initial inoculum concentration.

### Validation

The model was further validated using growth data at an unvalidated temperature of 10°C (Table 3). At 10°C, the observed lag time (LT) was 82.46±6.03 hours in LEW, 36.94±1.61 hours in LEY, and 48.79±0.67 hours in LWE, while the predicted LT values were 58.27 hours in LEW, 41.28 hours in LEY, and 49.61 hours in LWE. The observed *μ*_max_ was 0.008±0.005 in LEW, 0.034±0.001 in LEY, and 0.048±0.002 in LWE, whereas the predicted values were −0.111 in LEW, 0.033 in LEY, and 0.056 in LWE. In the LEW, *S.* Thompson growth remained restricted across temperature variations, leading to discrepancies between the predicted and observed values. However, in LEY and LWE, the predicted values closely matched the observed data, demonstrating the reliability of the model for estimating the growth of *S.* Thompson. Therefore, the growth prediction model for *S.* Thompson could serve as a valuable tool for determining the appropriate storage temperatures during the distribution of unpasteurized liquid egg products; these findings will contribute towards improved food safety management.

## CONCLUSION

In the present study, we investigated the growth kinetics of *S.* Thompson in LEWs, LEYs, and LWEs; we also developed and validated a predictive growth model using mathematical modeling. A uniform initial cell density of 5 log CFU/mL was inoculated and growth changes were monitored over time at various storage temperatures. The prediction model was developed on the basis of the Baranyi–Roberts model and a polynomial second-order model; we validated the model with regard to specific performance parameters.

In LEWs, *S.* Thompson exhibited limited growth, with a maximum increase of 1.0 log CFU/mL as the temperature increased. In contrast, rapid growth was observed in LEY and LWE, with increases of up to 4.0 log CFU/mL. The Baranyi–Roberts model served as the foundational model; we described the growth with regard to the LT and *μ*_max_ as temperature-dependent parameters.

A polynomial second-order model was applied to further analyze the temperature-dependent growth changes. Model performance was evaluated using the bias factor (B*_f_*), accuracy factor (A*_f_*), and RMSE. The validation results indicated a strong predictive accuracy, with B*_f_* and A*_f_* values close to 1 and low RMSE values. Additionally, model verification through a comparison of the predicted and observed values demonstrated a close alignment, confirming the reliability of the model.

This study has some limitations that should be considered when interpreting the results. First, the model was developed and validated using a single *S*. Thompson strain at one initial inoculum level, so it should be regarded as strain- and condition-specific rather than universally applicable. Second, the experiments were performed with unpasteurized commercial liquid egg products from a limited number of sources under isothermal conditions, which may not fully represent the variability in product characteristics and temperature fluctuations in practice. Further studies using multiple strains, diverse egg products, and non-isothermal conditions are needed to broaden the applicability of the model.

The growth risks of *S.* Thompson, which was responsible for a large-scale food poisoning outbreak in Korea [3], remain understudied. The predictive model developed in the present study effectively captures the complex growth dynamics of *S.* Thompson, demonstrating its high reliability as a forecasting tool. This model provides valuable insights into *S.* Thompson behavior, allowing for proactive risk management in food safety.

By accurately predicting the growth of *S.* Thompson, our findings can contribute towards enhancing HACCP plans and food safety management systems. Finally, our study will support food safety efforts by enabling the production of safer and more reliable food products.

## Figures and Tables

**Table 1:** Growth parameters of *Salmonella* Thompson in unpasteurized liquid egg whites, liquid egg yolks, and liquid whole eggs at different temperatures, as assessed based on the Baranyi–Roberts model

Temperature (°C)	LT (h)	*μ*_max_ (log CFU/mL/h)	*B* *_f_*	*A* *_f_*	RMSE	R^2^
Liquid egg white						
5	NO	−0.274±0.367^a^	1.0001	1.0118	0.0659	0.986
15	35.29±13.69^a^	0.009±0.002^b^	1.0003	1.0149	0.1008	0.646
25	7.24±0.79^b^	0.201±0.126^bc^	1.0005	1.0196	0.1391	0.785
35	2.97±0.35^b^	0.212±0.139^c^	1.0009	1.0217	0.1463	0.646
Liquid egg yolk						
5	60.79±5.48^a^	−0.003±0.015^a^	1.0005	1.0204	0.1314	0.676
15	25.63±3.79^b^	0.079±0.022^b^	1.0007	1.0267	0.1917	0.962
25	6.51±0.12^c^	0.478±0.020^c^	1.0009	1.0247	0.1952	0.966
35	2.74±0.25^d^	0.886±0.048^d^	1.0032	1.0328	0.2766	0.946
Liquid whole egg						
5	73.36±8.55^a^	−0.004±0.002^a^	1.0001	1.0084	0.0538	0.520
15	30.40±5.52^b^	0.065±0.012^b^	1.0008	1.0226	0.1503	0.973
25	7.39±0.29^c^	0.603±0.020^c^	1.0020	1.0324	0.2541	0.959
35	2.26±0.12^d^	0.862±0.023^d^	1.0027	1.0191	0.1543	0.986

The means of three replicates are plotted with the error bars representing the standard deviation.

a–d Means with the same lowercase letter within a row for the same sample are not significantly different (p<0.05).

LT, lag time; *μ*_max_, specific growth rate; *B_f_*, bias factor; *A_f_*, accuracy factor; RMSE, root mean square error; NO, no observation.

**Table 2:** Secondary model equation for predicting *Salmonella* Thompson growth in unpasteurized liquid egg products based on growth parameters

Sample	Parameter	Equation	R^2^
LEW	Polynomial model	*LT* = 122+(−7.562×*T*) + (0.1189×*T*^2^)	0.891
		*μ*_max_ = −0.4806+ (0.04374×*T*)+(−0.00068×*T*^2^)	0.577
LEY	Polynomial model	*LT* = 84.15+(−5.072×*T*)+(0.07849×*T*^2^)	0.986
		*μ*_max_ = −0.0286+(−0.002×*T*)+(0.00082×*T*^2^)	0.986
LWE	Polynomial model	*LT* = 101.6+(−6.145×*T*)+(0.09455×*T*^2^)	0.978
		*μ*_max_ = − 0.1151+(0.01232×*T*)+(0.00048×*T*^2^)	0.946

LEW, liquid egg white; LEY, liquid egg yolk; LWE, liquid whole egg.

**Table 3:** A comparison was made between the predicted and observed growth parameters of *Salmonella* Thompson in unpasteurized liquid egg whites (LEWs), liquid egg yolks (LEYs), and liquid whole eggs (LWEs) stored at 10°C

Sample		LT (h)	*μ*_max_ (log CFU/mL/h)	B*_f_*	A*_f_*	RMSE
LEW	Predicted value	58.27	−0.111	1.0003	1.0154	0.0992
	Observed value	82.46±6.03	0.008±0.005			
LEY	Predicted value	41.28	0.033	1.0008	1.0215	0.1437
	Observed value	36.97±1.61	0.034±0.001			
LWE	Predicted value	49.61	0.056	1.0001	1.0072	0.0439
	Observed value	48.49±0.67	0.048±0.002			

RMSE, root mean square error.

## Data Availability

Upon responsible request, the datasets of this study can be available from the corresponding author.
